# Predicting future medical needs and mortality risk in geriatric long-term care patients: Development and validation of the Nascher score and revised Nascher score

**DOI:** 10.1007/s00508-024-02410-9

**Published:** 2024-08-05

**Authors:** Thomas E. Dorner, Michael Smeikal, Matthias Unseld, Christoph Gisinger

**Affiliations:** 1 Academy for Ageing Research, Haus der Barmherzigkeit, Seeböckgasse 30a, 1160 Vienna, Austria; 2 Center for Public Health, Department for Social and Preventive Medicine, Medical University of Vienna, Vienna, Austria; 3 Karl Landsteiner Institute for Health Promotion Research, Haus der Barmherzigkeit Clementinum, Kirchstetten, Austria

**Keywords:** Gerontology, Risk of death, Assessment of medical care needs, Nursing hospital, Medical effort

## Abstract

**Background:**

Choosing the right intensity of medical care is a huge challenge particularly in long-term geriatric care. The Nascher score was developed to assess future medical care needs. The aim of this study was to determine whether the Nascher score and a revised version can predict future medical needs.

**Methods:**

In this retrospective cohort study, 396 residents in long-term care hospitals, who were admitted over a period of two years and followed up to two and a half yeare, were analysed. Outcome parameters were: (1) number of medication changes, (2) number of ward doctor documentations and (3) number of acute illnesses treated with antibiotics, and mortality risk. Based on the first results, an alternative scoring of the Nascher score with 12 instead of 26 items was developed, called the revised Nascher score.

**Results:**

The Nascher score significantly correlated with the number of medication changes, the number of ward doctor documentations, and the number of acute ilnesses treated with antibiotics with Spearman correlation coefficients of 0.30, 0.26, and 0.15, respectively. The revised Nascher score showed a higher correlation with correlation coefficients of 0.36, 0.26, and 0.21, respectively. Residents with a Nascher score in the highest quartile had a significantly higher mortality risk than residents in the lowest quartile (hazard ratio, HR 2.97, 95% confidence interval, CI 1.80–4.34). The corresponding values for the revised Nascher score were HR 3.03, 95% CI 2.03–4.54 in the highest and HR 1.80, 95% CI 1.24–2.60 in the middle quartiles.

**Conclusion:**

The Nascher score and even more so the revised Nascher score are well suited to predicting the various parameters of future medical needs and mortality risk.

## Introduction

One of the biggest challenges in caring for older people is choosing the right level of care [1]. Although many chronic diseases do not require permanent medical treatment, acute exacerbations, complications, and side effects of medical treatment, can occur at any time. Multimorbidity, in particular, is associated with unpredictable medical care needs [2]. Many people with chronic illnesses therefore require more medical care than can be provided in traditional nursing homes and less medical care than staying permanently in acute hospitals. For these people, it is important to be able to estimate their future medical care needs in order to ensure the right level of care according to the basic principle, as intensively as necessary and as extensively as possible.

Many geriatric assessment instruments are routinely used to record the resources and barriers of older people and to monitor them over time. In the geriatric assessment, particular emphasis is placed on recording the resources and impairments that people usually do not speak about on their own. Instruments have been established in geriatric assessment to assess, for example, nursing care needs, cognitive function, depression, ability to cope with activities of daily living, mobility, risk of falls, nutritional status and risk of developing a pressure sore or frailty [3–6]. Applying geriatric assessment has been shown to be very helpful in determining the right level of care [7]; however, to date no instrument has been established in geriatric assessment to assess the expected medical needs. This was an important reason for the development of the Nascher score.

The Nascher score is named after the Austrian-American geriatric pioneer and founder of the term “geriatrics”, Ignatz Leo Nascher [8]. It was developed in 2007 and has been routinely applied to all new residents of the Haus der Barmherzigkeit long-term care hospitals in Vienna, Austria, which provide advanced nursing care, rehabilitative therapy and also comprehensive medical care with physicians on duty 24/7. The Nascher score includes four domains defined as geriatric patient, risks for medical incidents, the application of special medical measures and therapies, and advanced chronic diseases. To date, a systematic evaluation or validation of the Nascher score has not been conducted.

The aim of this study was to determine whether the Nascher score can actually predict future medical needs and mortality risk. Another aim was to determine whether a shorter version of the Nascher score using an alternative scoring system was possible without compromising validity.

## Methods

The analysis was performed as a retrospective cohort study with the routine medical recordings of residents of the two nursing hospitals of Haus der Barmherzigkeit in Vienna, Austria. All residents of the nursing hospitals who were admitted between 1 August 2020 and 31 July 2022 were included (in total 529 cases). Those who had already been admitted to the Haus der Barmherzigkeit hospitals at least once in the previous 2 years and those who it had been planned to admit but were never in fact admitted were excluded. The analyzable cohort ultimately consisted of a total of 507 people. Patients with other reasons for admission other than long-term care (such as short-term care or rehabilitation) and subjects with no Nascher score at the time of admission were excluded, yielding in a sample size of 396 persons. The observation period was the respective time of admission until discharge, death, or 31 May 2023, whichever occurred first.

**Fig. 1:**
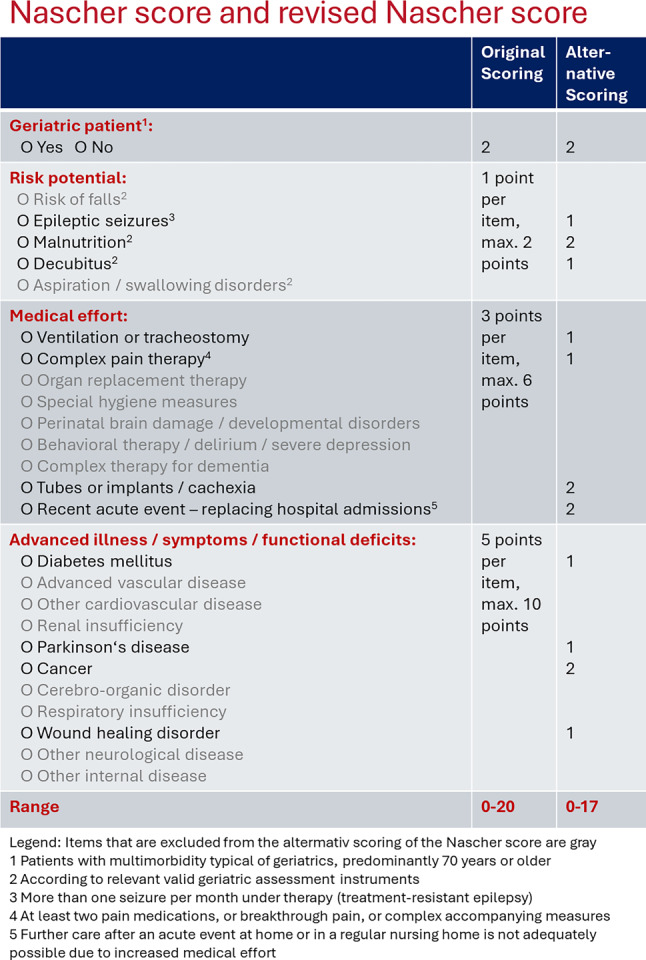
Items of the Nascher score. (original and alternative scoring)

The Nascher score is routinely applied before or during admission of residents by a medical doctor, typically by the medical director of the nursing hospital or a deputy. The parameters of the Nascher score are taken manually from the electronic medical documentation charts and transformed into a case report form and then entered into statistical software. In this study, if several Nascher scores were available for an individual, the score that was calculated after admission and was closest to the day of admission was used. Firstly, all the analyses were calculated using the Nascher score with the original scoring system used since its creation (see Fig. 1). Based on these results, an alternative system for the revised Nascher score was established, applying the following procedure: individual items that occurred very rarely (< 5% of residents) were removed from the Nascher score. Furthermore, individual items that showed an inverse association with medical needs were also removed. Finally, all the individual items that showed a significant association with the outcomes were rated with two points and those that showed a lesser association with the outcomes with one point (see Fig. 1). The results of the Nascher score and the revised Nascher score were calculated using statistical software. The original Nascher score consists of 26 items, and the score has a range from 0 to 20 points. The revised Nascher score consists of 12 items and has a range from 0 to 17 points. Higher scores mean higher anticipated medical needs. For some of the analyses, the Nascher score and the revised Nascher score were categorized according to their quartiles into three categories, where the middle two quartiles were merged into one category.

Sex (male or female), age in years, and level of care were obtained from the electronic medical documentation system. The level of care was measured by the level of Austrian long-term care allowance, which is paid to all Austrian residents with a disability expected to last longer than 6 months, in seven different levels of care (*Pflegestufen*), according to their needs of care. A level of 3 or higher is the prerequisite for nursing home or long-term care nursing hospital admission [9]. For some analyses, age and level of care were dichotomized using a median split.

The primary outcome was the need for medical care. This was operationalized by: (1) the number of medication changes per year, (2) the number of ward doctor documentations per week and (3) the number of acute illnesses treated with antibiotics per year. All three outcome parameters were also taken from the electronic medical charts. An antibiotic episode was defined as at least one administration of antibiotics being documented on one or more subsequent days. If no antibiotic was given for at least two consecutive days and then antibiotic therapy was restarted, this was counted as two episodes treated with antibiotics. Furthermore, the occurrence of death was defined as a further additional outcome parameter.

Measures for the distribution of variables skewness and excess kurtosis were computed. A normal distribution has a skewness value and an excess kurtosis value of 0. A distribution with a positive skewness value is right-skewed, and a distribution with a negative skewness value is left-skewed. A positive excess kurtosis value indicates that the data contain more extreme outliers than a normal distribution, and negative kurtosis means that the data has fewer extreme outliers than a normal distribution. Descriptive analyses in the form of frequency tables and the mean, standard deviation, median, interquartile range, and minimum and maximum were used in the statistical evaluations. Furthermore, we applied correlation analyses (two-time Spearman correlation coefficients), cross-tabulation with the χ^2^-test, and for comparisons of medians we used non-parametric tests. In the Cox regression models and Kaplan-Meier curves, the factors influencing mortality risk were calculated. As the censoring factor, discharge from the hospital, death, or the end of the observation period was applied, whichever occurred first. The results of the Cox regression models are presented as the hazard ratio (HR) and 95% confidence interval (95% CI). A HR > 1 can be interpreted as a positive relationship and an HR < 1 as an inverse relationship. If the CIs do not overlap, this is considered a significant difference. All the statistical analyses were carried out using a professional statistics software.

The study protocol was discussed at a meeting of the Advisory Committee for Ethical Issues in Scientific Studies at Haus der Barmherzigkeit on 20 June 2023 and approved. The data were analyzed in pseudonymized form (the pseudonyms were the respective admission numbers).

## Results

The characteristics of the 396 included individuals are shown in Table 1. There were slightly more women than men, the mean age was 81 years, and the mean care level was 4. Only a few residents were discharged home or to a hospital, and most of the residents either died in the nursing hospital or were still living in the nursing hospital at the end of the observation period. The mean Nascher score was 17 points in the original scoring system and 6 points in the alternative scoring system. The results of the original scoring system showed a very left-skewed distribution (skewness: −1.21; excess kurtosis: 1.89), while the results of the alternative scoring system were normally distributed (skewness: 0.27; excess kurtosis: 0.05). The residents had a median of 16.3 medication changes per year, 1.8 ward doctor documentations per week and 1.5 acute illnesses treated with antibiotics per year (all three parameters showed a very right-skewed distribution).

**Table 1:** Characteristics of the 396 included participants in the geriatric long-term care hospitals

*Sex*	
Male	172 (43.4%)
Female	224 (56.6%)
*Age in years*	
Mean (SD)	81.2 (10.8)
Median (IQR)	82.2 (74.7; 89.2)
Range	40.5–103.3
*Level of care on day of admission*	
Mean (SD)	4.2 (1.4)
Median (IQR)	4 (3; 5)
Range	0–7
*Duration of observation (days)*	
Mean (SD)	334 (291)
Median (IQR)	291 (59; 545)
Range	1–1031
*Reason for termination of observation*	
Resident until end of observation period	150 (37.9%)
Discharge home	29 (7.3%)
Admission to hospital	5 (1.3%)
Discharge to other nursing home	8 (2.0%)
Remained in the nursing hospital until death	175 (44.2%)
Died in hospital	29 (7.3%)
*Nascher score (original scoring)*	
Mean (SD)	16.8 (3.2)
Median (IQR)	17 (15; 20)
Range	3–20
*Nascher score (alternative scoring)*	
Mean (SD)	5.9 (2.4)
Median (IQR)	6 (4; 7)
Range	0–14
*Number of medication changes per year*	
Mean (SD)	40.6 (54.6)
Median (IQR)	16.3 (9.0; 45.8)
Range	0–296.6
*Number of ward doctor documentations per week*	
Mean (SD)	2.6 (2.5)
Median (IQR)	1.8 (1.0; 3.2)
Range	0–20.3
*Number of acute illnesses treated with antibiotics*	
Mean (SD)	4.2 (7.6)
Median (IQR)	1.5 (0; 4.7)
Range	0–52.1

All numbers are number of participants (%), unless other specified

*SD* standard deviation, *IQR* interquartile range

The most common parameters of the Nascher score were geriatric patient (92.2%), recent acute event (82.1%), risk of falls (79.8%), advanced vascular disease (62.6%), other cardiovascular disease (42.2%), other internal disease (41.4%), and malnutrition (32.8%). A diagnosis of cancer was found in 25.8% of participants, and tubes, implants, or cachexia were documented in 19.2% of participants.

The Nascher score correlated significantly with the three outcomes, with Spearman correlation coefficients of 0.30 (*P* < 0.001), 0.26 (*P* < 0.001), and 0.15 (*P* = 0.002) for the number of medication changes per year, the number of ward doctor’s documentations per week, and the number of acute illnesses treated with antibiotics per year, respectively. Individual parameters of the Nascher score that were particularly strongly associated with the outcome parameters were geriatric patient, malnutrition, presence of tubes, implants, or cachexia, recent acute events and malignancies. These parameters were then rated higher in the alternative scoring. The revised Nascher score showed a slightly better correlation with the outcome parameters, with Spearman correlation coefficients of 0.36, 0.26, and 0.21, respectively for the three outcome parameters (*P* < 0.001 for each parameter).

Table 2 lists the median values for medical needs according to the Nascher score categories. There was a clear significant and gradual association between the Nascher score on admission to the nursing hospital and the medical effort required in the following months for all three outcome parameters. This gradual association could be observed for both the original and alternative scoring systems of the Nascher score; however, with the alternative scoring of the revised Nascher score, the differences in the medians of the outcome categories were more pronounced than with the original scoring.

**Table 2:** Median values for outcomes according to the Nascher categories and the revised Nascher categories

	Median number of medication changes per year	Median number of ward doctor documentations per week	Median number of acute illnesses treated with antibiotics per year
*Original scoring of the Nascher score*			
Lowest quartile (0–14 points)	10.2	1.32	0.90
Middle quartiles (15–19 points)	14.9	1.69	1.42
Highest quartile (20 points)	33.7	2.57	2.21
P	< 0.001	< 0.001	0.003
*Alternative scoring of the revised Nascher score*			
Lowest quartile (0–4 points)	10.1	1.44	0.88
Middle quartiles (5–7 points)	17.1	1.83	1.84
Highest quartile (8–14 points)	42.1	2.78	2.21
P	< 0.001	< 0.001	< 0.001

**Fig. 2:**
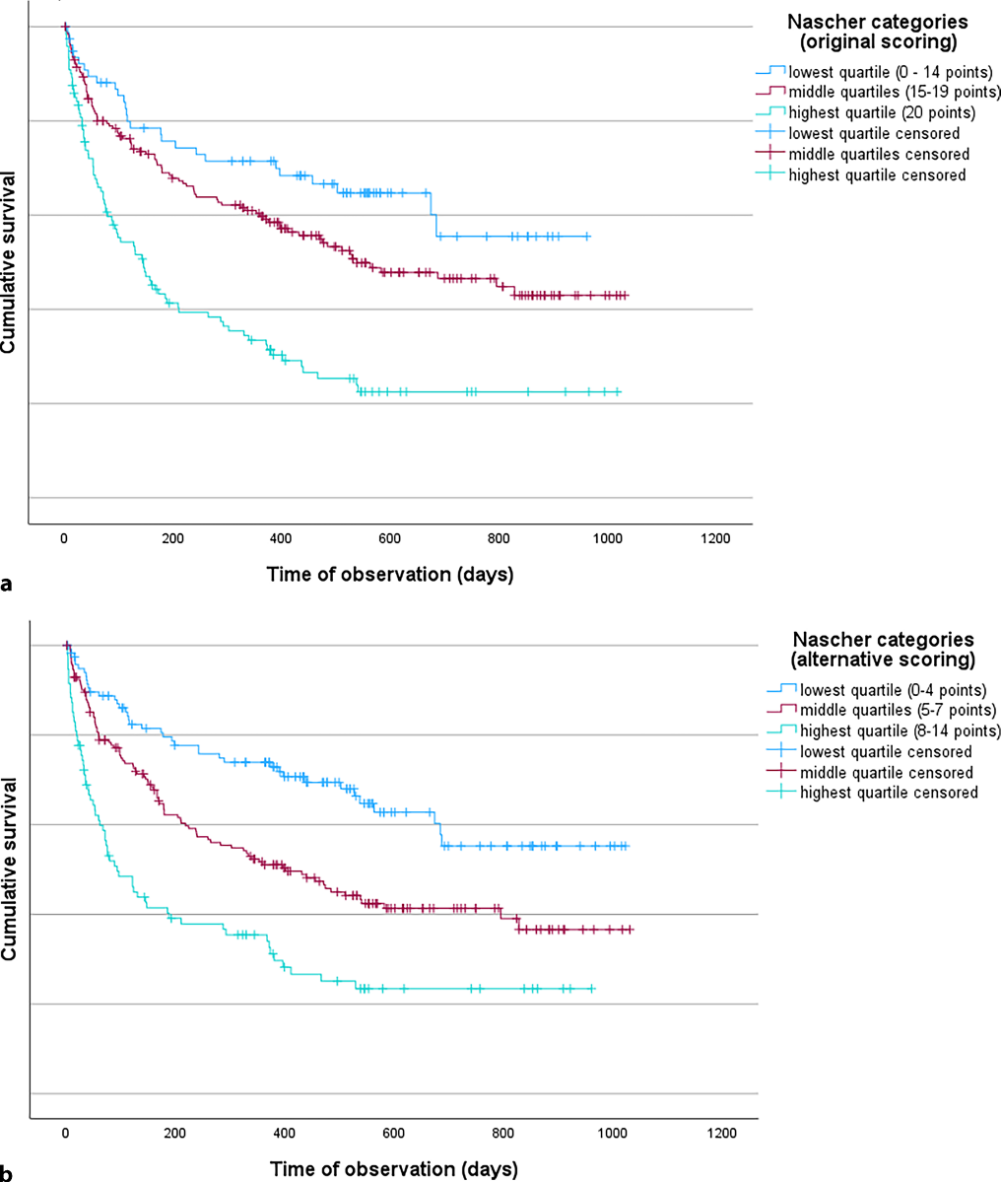
Kaplan-Meier survival curves of geriatric long-term nursing hospital residents depending on the Nascher score categories. **a** Original scoring, **b** alternative scoring

In total, 204 participants (51.5%) died during the observation period. Figure 2a shows the survival rates by Nascher score category, using the original scoring system. The higher the Nascher score, the lower the probability of survival. In the highest quartile of the Nascher score, 70.0% died during the observation period, compared to 47.0% in the middle quartiles and 34.6% in the lowest quartile (*P* < 0.001). Residents with a Nascher score in the highest quartile had a median survival time of 145 days (95% CI: 88–201 days), compared to 538 days (95% CI: 279–797 days) in the middle quartiles and a medium survival beyond the observation time in the lowest quartile (more than half of the participants in these quartiles still lived at the end of the observation period). Similar findings could be observed with the revised Nascher score (Fig. 2b). Here, in the highest quartile of the Nascher score 70.2% died during the observation period compared to 53.3% in the middle quartiles and 33.9% in the lowest quartile (*P* < 0.001). Participants with a Nascher score in the highest quartile had a median survival time of 93 days (95% CI: 43–143 days) compared to 401 days (95% CI: 253–548 days) in the middle quartiles and a medium survival beyond the observation time in the lowest quartile.

The factors influencing the risk of death are shown in Table 3. Even after adjusting for age, sex, and level of care, a higher Nascher score was significantly associated with a higher mortality risk. A Nascher score in the highest quartile increased the mortality risk threefold, compared to the lowest quartile. Age, sex, and level of care showed no significant association with mortality risk. The hazard ratios were slightly more pronounced with the revised Nascher score than with the original scoring system.

**Table 3:** Factors associated with the risk of death in long-term care nursing hospitals. Results of the Cox regression analyses. All parameters are mutually adjusted for the others

	Original scoring of the Nascher score		Alternative scoring of the revised Nascher score	
	HR	95% CI	HR	95% CI
*Age*				
Lower than median (81.8 years)	1		1	
Higher than median	1.24	0.75–1.42	1.23	0.93–1.64
*Sex*				
Female	1		1	
Male	1.28	0.96–1.70	1.25	0.94–1.67
*Level of care*				
Lower than median (1–3)	1		1	
Higher than median (4–7)	1.03	0.75–1.42	1.02	0.74–1.67
*Nascher score/revised Nascher score*				
Lowest quartile (0–14/0–4)	1		1	
Middle quartiles (15–19/5–7)	1.42	0.92–2.18	1.80	1.24–2.60
Highest quartile (20/8–14)	2.79	1.80–4.34	3.03	2.03–4.54

*HR* hazard ratio, *CI* confidence interval

## Discussion

In this cohort study the relationships between the Nascher score, which has been developed and used for routine purpose since 2007, and the various outcome parameters of medical needs and risk of death were analyzed. Based on the findings of a first analysis with the original scoring system, an alternative scoring system for the Nascher score was established. There was a significant correlation between the Nascher score upon admission to the nursing hospital and the number of medication changes per year, the number of ward doctor documentations per week, and the number of episodes treated with antibiotics per year. Furthermore, a higher Nascher score significantly predicted a higher mortality risk, even given age, sex, and level of care.

Both the Nascher score and the revised Nascher score showed very good predictive power for medical needs, with the revised Nascher score performing slightly better. Furthermore, the revised Nascher score has several advantages over the original version: (1) fewer parameters are required to perform the scoring (12 vs. 26 items) and therefore it is significantly less time-consuming and (2) the revised Nascher score is normally distributed. This implies the advantage of applying parametric statistical methods, which are more powerful and versatile than the non-parametric alternatives required for skewed data. This enhances the reliability and validity of the assessment tool by enabling precise comparisons across different groups and time points. Moreover, a normal distribution ensures that extreme scores are proportionately represented, preventing the distortion of results that could lead to inaccurate interpretation. For instance, the use of normally distributed scores in clinical assessments has been shown to improve the precision of diagnostic tools and the effectiveness of therapeutic interventions [10].

Surprisingly, to date the assessment of medical needs has received little attention in geriatric assessment. While future medical care needs are usually not specifically addressed in routine geriatric assessments, they often include elements that indirectly address future health. This includes evaluating risk factors for falls, frailty, or common geriatric conditions, which then individually and through their interaction determine the medical needs [11]. Therefore, the Nascher score could be viewed as a shortcut to assess medical expenditure. Furthermore, planning for future medical needs is often addressed through other services and discussions, such as advanced care planning, palliative care consultation, and long-term care planning [12]. The Nascher score could help with making decisions about nursing and medical care pathways, especially with respect to medical care, including whether the presence of physicians is needed around the clock or not.

Some items of the Nascher score were particularly strongly associated with future medical needs, i.e., designation as geriatric patient, malnutrition, presence of tubes, implants, or cachexia, recent acute events, and malignancies. Malnutrition is, along with sarcopenia and chronic inflammation, the most important factor involved in the development of frailty. Furthermore, frailty is a geriatric syndrome that predicts many negative outcomes [13]. In addition, malnutrition is often the result of many chronic diseases that are consumptive and involve loss of appetite, difficulty chewing or swallowing, or malabsorption [14], and malnutrition predicts many adverse health outcomes [15]. Very often cachexia is a result of malnutrition and requires oral supplementation or enteral nutrition [16]. It is therefore not surprising that malnutrition and also the presence of tubes, implants, or cachexia predict medical needs so strongly. Cancer was also a diagnosis that very strongly predicted medical needs. Medical needs in patients, even after acute cancer treatment, remain high and include care for long-term side effects and complications of surgery, chemotherapy, radiotherapy and immunotherapy, and life-long therapies such as hormone replacement, but also surveillance for recurrence and secondary cancers, chronic health conditions, and mental and psychological support. These are also reasons why patients with cancer significantly benefit from routine geriatric assessments [17, 18].

To evaluate the Nascher score, two apparently very different outcome clusters were used: medical effort and death; however, the time just before death is often a medically very complex and intensive time [19]. This was also shown by a recent analysis with data from the same cohort [20]. Therefore, it is not surprising that the Nascher score results are similar with respect to both outcome clusters. About half of the patients included in the study died during the observation period, a median of 3 months after admission.

Notably, the level of nursing care at admission was not a predictor of death, in contrast to the Nascher score. This underlines that in geriatric care medical care needs and nursing care needs are two distinct concepts, each addressing different aspects of an older adult’s health. An integrative approach is essential where medical care and nursing care complement each other to address the comprehensive needs of older adults [21, 22]. In this sense, applying the Nascher score could very well complement assessing nursing care needs in terms of medical care needs.

Assessing future medical needs in geriatric patients is important for optimizing patient outcomes and resource allocation. Geriatric patients often present with complex, multimorbid conditions that require ongoing, multidimensional assessments and individualized interdisciplinary care plans to be managed effectively [23, 24]. Medical staff in long-term care facilities are uniquely positioned to implement and monitor health conditions, enabling a proactive approach to healthcare that is responsive to the nuanced needs of the residents. This continuous assessment framework supports the integration of preventive strategies, personalized treatment plans, and coordinated care efforts, thereby improving overall health outcomes and operational efficiency within these facilities [25].

The strength of this study is that it is the first analysis of the predictive power of an instrument that has been used for many years to assess medical needs in geriatric long-term care nursing hospital residents. This was analyzed using a cohort of all the people who were admitted to the nursing hospitals of Haus der Barmherzigkeit over a 2-year period, with an observation period of up to 2.5 years. Conscientious and complete electronic documentation over many years was a prerequisite to make this analysis possible. A possible limitation inherent in the retrospective nature of this study is that the outcome parameters were recorded for the continuous documentation of the medical needs and not primarily with the intention of conducting a scientific study. To date, the Nascher score has only been used in geriatric long-term care hospitals in two locations. The analysis was performed with a limited sample size of 396 subjects. Therefore, the generalizability to other settings is limited. Another limitation is that no data for sensitivity or specificity are yet available, and no comparisons with other clinical scores were performed. In the future, the Nascher score could be further developed, including possible changes to the categorization, application of the Nascher score in other healthcare facilities, and observations over a longer period of time.

In conclusion, it can be said that the Nascher score and even more so the revised Nascher score are well suited to predict the various parameters of future medical needs. Furthermore, a higher Nascher score is associated with a higher mortality risk. The Nascher score can be used to determine the adequate level of medical care in geriatric patients by discriminating between those who require more or less medical care and can therefore be added to routine evaluation of nursing needs and geriatric assessment.
